# Targeting CD44-STAT3 Signaling by Gemini Vitamin D Analog Leads to Inhibition of Invasion in Basal-Like Breast Cancer

**DOI:** 10.1371/journal.pone.0054020

**Published:** 2013-01-11

**Authors:** Jae Young So, Amanda K. Smolarek, David M. Salerno, Hubert Maehr, Milan Uskokovic, Fang Liu, Nanjoo Suh

**Affiliations:** 1 Department of Chemical Biology, Ernest Mario School of Pharmacy, Rutgers, The State University of New Jersey, Piscataway, New Jersey, United States of America; 2 Graduate Program in Cellular and Molecular Pharmacology, Rutgers, The State University of New Jersey, Piscataway, New Jersey, United States of America; 3 Center for Advanced Biotechnology and Medicine, Rutgers, The State University of New Jersey, Piscataway, New Jersey, United States of America; 4 The Cancer Institute of New Jersey, New Brunswick, New Jersey, United States of America; National University of Ireland Galway, Ireland

## Abstract

**Background:**

CD44, a transmembrane glycoprotein, is a major receptor for extracellular proteins involved in invasion and metastasis of human cancers. We have previously demonstrated that the novel Gemini vitamin D analog BXL0124 [1α,25-dihydroxy-20R-21(3-hydroxy-3-deuteromethyl-4,4,4-trideuterobutyl)-23-yne-26,27-hexafluro-cholecalciferol] repressed CD44 expression in MCF10DCIS.com basal-like human breast cancer cells and inhibited MCF10DCIS xenograft tumor growth. In the present study, we investigated potential factors downstream of CD44 and the biological role of CD44 repression by BXL0124 in MCF10DCIS cells.

**Methods and Findings:**

The treatment with Gemini vitamin D BXL0124 decreased CD44 protein level, suppressed STAT3 signaling, and inhibited invasion and proliferation of MCF10DCIS cells. The interaction between CD44 and STAT3 was determined by co-immunoprecipitation. CD44 forms a complex with STAT3 and Janus kinase 2 (JAK2) to activate STAT3 signaling, which was inhibited by BXL0124 in MCF10DCIS cells. The role of CD44 in STAT3 signaling and invasion of MCF10DCIS cells was further determined by the knockdown of CD44 using small hairpin RNA *in vitro* and *in vivo*. MCF10DCIS cell invasion was markedly decreased by the knockdown of CD44 *in vitro*. The knockdown of CD44 also significantly decreased mRNA expression levels of invasion markers, matrix metalloproteinases (MMPs) and urokinase plasminogen activator (uPA), in MCF10DCIS cells. In MCF10DCIS xenograft tumors, CD44 knockdown decreased tumor size and weight as well as invasion markers.

**Conclusions:**

The present study identifies STAT3 as an important signaling molecule interacting with CD44 and demonstrates the essential role of CD44-STAT3 signaling in breast cancer invasion. It also suggests that repression of CD44-STAT3 signaling is a key molecular mechanism in the inhibition of breast cancer invasion by the Gemini vitamin D analog BXL0124.

## Introduction

Invasive growth is a physiological property of embryonic cells during development and epithelial cells during wound healing [1], [2]. However, under pathological conditions, invasive growth of cancer cells is one of the hallmarks of malignancy progression evidenced by local invasion and distant metastasis [3]. In breast cancer, ductal carcinoma in situ (DCIS) has been recognized as a precursor of invasive ductal carcinoma (IDC) [4]. The acquisition of an invasive phenotype has been suggested to be a critical step in the transition from DCIS to IDC [4], [5], [6]. However, many studies failed to elucidate the complex nature of the DCIS to IDC transition [4]. Recent studies demonstrated that both cancer cells and the tumor-associated microenvironment, such as extracellular matrix and stromal cells, are critical contributors to cancer invasion [3], [6], [7]. These findings highlight the importance of molecules involved in microenvironment-epithelial interactions as potential therapeutic targets.

CD44 is one of the key molecules that regulate microenvironment-epithelial interactions by serving as a major receptor for several extracellular matrix proteins such as hyaluronan and osteopontin [8]. CD44 overexpression correlates with invasive and metastatic phenotype in breast cancer, and thus, is an indicator of poor prognosis [9], [10]. Recently, CD44 has been recognized as one of the key cell surface markers for tumor-initiating cells in breast cancer [11], [12]. Since CD44 does not have intrinsic kinase activity, it modulates intracellular signaling by interacting with other components of signaling transduction such as receptor tyrosine kinases or intracellular kinases [8], [13]. The recruitment of signaling partners and resulting signaling by CD44 depends on the types of microenvironment and tumors [13]. Therefore, identification of interacting molecules in a cell-type specific manner is important to understand the biological role of CD44 in human breast cancer.

Signal transducer and activator of transcription 3 (STAT3) is a transcription factor that mediates the cellular response to various cytokines and growth factors [14]. Upon activation, STAT3 is phosphorylated by intracellular kinases, including Janus kinase 2 (JAK2) and Src [14]. In human cancers, including breast cancer, the persistent activation of STAT3 is often associated with tumor progression [15], [16]. Studies with human breast cancer cells demonstrated that the constitutive activation of STAT3 is a crucial contributor to the growth, survival and invasion of cancer cells [17], [18]. On the other hand, inhibition of STAT3 signaling with STAT3 small hairpin RNA (shRNA) or use of STAT3 phosphorylation inhibitors repressed the formation and growth of xenograft tumors in mice as well as the invasive potential of breast cancer cells [19], [20]. A recent study reported that CD44^+^ tumor-initiating breast cancer cells had preferential activation of STAT3, suggesting that STAT3 may be a potential therapeutic target in breast cancer [21].

We have previously shown that a novel Gemini vitamin D analog, BXL0124, down-regulated CD44 expression in MCF10DCIS cells and inhibited tumor growth in a MCF10DCIS xenograft [22]. However, the biological role of CD44 repression by BXL0124 in breast cancer has not been fully explored. In the present study, we investigated the effect of BXL0124 on key molecules in various signaling pathways and invasion of MCF10DCIS cells. We demonstrate that the repression of CD44 by BXL0124 contributes to the inhibition of STAT3 signaling and tumor invasion in MCF10DCIS cells.

## Materials and Methods

### Reagents and Cell Culture

1α,25(OH)_2_D_3_ and Gemini vitamin D analog 1α,25-dihydroxy-20R-21(3-hydroxy-3-deuteromethyl-4,4,4-trideuterobutyl)-23-yne-26,27-hexafluro-cholecalciferol (BXL0124, [23]) were provided by BioXell, Inc. (Nutley, NJ) and dissolved in dimethyl sulfoxide. The MCF10DCIS.com and MCF10CA1a human breast cancer cell lines were provided by Dr. Fred Miller at the Barbara Ann Karmanos Cancer Institute (Detroit, MI) [24], [25]. The MCF10DCIS.com cell line was authenticated by short tandem repeat profiling at American Type Culture Collection (Manassas, VA). MCF10DCIS-shLuc and MCF10DCIS-shCD44 cells were generated by infecting the MCF10DCIS.com cells with lentivirus encoding shRNA to luciferase (shLuc) or shRNA to CD44 (shCD44) [12]. The infected cells were sorted by FACS through the green fluorescence protein (GFP) to obtain GFP-labeled DCIS-shLuc cells or GFP-labeled DCIS-shCD44 cells. Cells were maintained in DMEM/F-12 medium supplemented with 5% horse serum, 1% penicillin/streptomycin, and 1% HEPES solution at 37°C and 5% CO_2_. MDA-MB-468 human breast cancer cells were cultured in DMEM/F-12 medium supplemented with 10% fetal bovine serum and 1% penicillin/streptomycin at 37°C and 5% CO_2_.

### [^3^H]Thymidine Incorporation Assay

The procedure was described previously [26]. In brief, MCF10DCIS cells seeded into a 24-well plate (8,000 cells per well) were treated next day with given doses of 1α,25(OH)_2_D_3_ or BXL0124 for 72 h. DCIS-shLuc or DCIS-shCD44 cells (2,000 cells) were incubated for 48 h after seeding for the thymidine incorporation assay.

### MTT Assay

MCF10DCIS cells seeded into a 96-well plate (1,000 cells/well) were treated on the next day with 0,01, 0,1, 1, 10 or 100 nM of 1α,25(OH)_2_D_3_ or BXL0124 for given incubation times. At each time point, 10 µl of MTT-I solution (thiazolyl blue tetrazolium bromide, M2128, Sigma-Aldrich, St. Louis, MO) were added into each well and incubated for 5 h, followed by the addition of 100 µl of MTT-II solution (distilled water with10% SDS and 0.01 M HCl). The plate was then incubated overnight, and the absorbance was measured with a spectrophotometer (Tecan US, Durham NC) at 560 nm.

### Cancer Cell Invasion Assays

Three different cell invasion assays were used: 3D culture assay with Matrigel (BD Bioscience, Spark, MD), Cultrex®24 well basement membrane extract (BME) cell invasion assay (Trevigen, Gaithersburg, MD), and Fluoroblok Biocoat cell invasion assay (BD Bioscience, Sparks, MD). For 3D culture, 4-well culture slides, coated with Matrigel, were prepared as previously described [27]. MCF10DCIS cells were seeded as single cells in M171 mammary epithelial medium (Invitrogen, Carlsbad, CA) supplemented with mammary epithelial growth supplement (Invitrogen, Carlsbad, CA). The cells were incubated for 10 days, and medium was replenished every 2 days. Cultrex®24 well BME cell invasion assay and Fluoroblok Biocoat cell invasion assay were performed as described in the manufacturers’ protocols. In the Cultrex®24 well BME cell invasion assay, the cells that penetrated matrigel were dissociated from the bottom of chamber and stained with Calcein-AM as described in the manufacturer’s protocol. The intensity of Calcein-AM fluorescence was measured by a fluorescent plate reader (Tecan US) and compared to a pre-measured standard curve to determine the number of cells per well. For quantitative evaluation of Fluoroblok Biocoat cell invasion assay, the green pixel counts per total pixel counts from 4 representative pictures per well were calculated using the Image J program (NIH, Bethesda, MD) (http://rsbweb.nih.gov/ij).

### Quantitative Real-time Polymerase Chain Reaction

The procedure was described previously [26]; the labeled primers for CD44, matrix metalloproteinase (MMP)-2, MMP-9, MMP-13, MMP-14, MMP-15, MMP-16, tissue inhibitor of metalloproteinase (TIMP)-1, TIMP-2, uPA, and glyceraldehyde 3-phosphate dehydrogenase were obtained from Applied Biosystems (Foster City, CA).

### Western Blot Analysis

The detailed procedure was described previously [28]. The primary antibody against CD44 (sc-7298), which recognizes both CD44v and CD44s, was from Santa Cruz Biotechnology (Santa Cruz, CA). Primary antibodies recognizing pSTAT3 (9235), STAT3 (9139), pAkt (9271), Akt (2966), pErk (9101) and Erk (9109) were from Cell Signaling Technology (Beverly, MA); pNFκB (sc-101749) and NFκB (sc-372) were from Santa Cruz Biotechnology; MMP-9 (ab38898) was from Abcam (Cambridge, MA); vitamin D receptor (VDR) (GR37) was from Millipore (Billerica, MA); β-actin (A1978) was from Sigma-Aldrich (St. Louis, MO). Secondary antibodies were from Santa Cruz Biotechnology.

### Knockdown of VDR by siRNA

The detailed procedure was described previously [22]. MCF10DCIS cells were incubated with 1 µM of non-targeting siRNA (D-001910-02-20, Thermo Fisher Scientific, Waltham, MA) or VDR siRNA (A-003448-13-0010, Thermo Fisher Scientific) for 72 h in Accell siRNA delivery medium (Thermo Fisher Scientific).

### STAT3 DNA Binding Assay

Transfactor STAT3-specific chemiluminescent kit from Clontech (Mountain View, CA) was utilized according to the manufacturer’s instructions. In brief, whole cell lysates of MCF10DCIS cells (40 µg) were incubated for 1 h in the Transfactor assay plates, which contained oligonucleotides with STAT3 binding sequences. STAT3 primary and secondary antibodies (provided with the kit) were incubated for 60 and 30 minutes, respectively. The mixture of chemiluminescent substrate A and B (1∶1) was added, and chemiluminescent intensity was measured by luminometer (Turner Biosystems, Sunnyvale, CA). The chemiluminescent intensity values of samples treated with BXL0124 were divided by the chemiluminescent intensity value of a control sample, and the fold changes were calculated.

### Fluorescence Microscopy

For *in vitro* samples, cells were fixed as previously described [22]. For *in vivo* samples, the tumors were embedded in paraffin (Electron Microscopy Sciences, Hatfield, PA) and then sectioned at 4 µm thickness. Both cell and tumor samples were incubated with PBS containing 10% goat serum to block non-specific binding. Fixed cells were incubated overnight at 4°C with a primary antibody to pSTAT3 (Cell Signaling Technology, 1∶500). Similarly, tumor samples were incubated with a combination of primary antibodies to pSTAT3 (Cell Signaling Technology, 1∶100) and CD44 (Santa Cruz Biotechnology, 1∶100). Fluorophore-conjugated secondary antibody (Alexa Fluor 488 or 546; Invitrogen, 1∶200) and TO-PRO-3 iodide nuclear antibody (Invitrogen, 1 µM) were incubated at room temperature for 60 and 15 minutes, respectively. The images were taken using confocal microscope with laser at 488 nm (pSTAT3), 546 nm (CD44), and 633 nm (TO-PRO-3).

### Immunoprecipitation

After 24 h incubation with or without BXL0124, MCF10DCIS cells were washed once with PBS and lysed in immunoprecipitation lysis buffer (Thermo Fisher Scientific). Antibodies to STAT3 or JAK2 (Cell Signaling Technology) were immobilized to protein G-conjugated Dynabeads (Invitrogen). The antibody-conjugated beads were washed by magnetic separation, and same amounts of protein samples were added. After a 10-minute incubation, the Dynabead-antibody-protein complex was isolated by magnetic separation and washed three times. Immunoprecipitated proteins were then detected by Western blot analysis.

### Xenograft Tumor Study

MCF10DCIS-shLuc or MCF10DCIS-shCD44 cells were injected into the mammary fat pad of immunodeficient nu/nu mice as described previously [29]. Tumor size was measured twice weekly. Five weeks after the cell injection, mice were sacrificed and xenograft tumors were weighed. The tumor samples were fixed in 10% formalin and transferred to 70% ethanol for immunofluorescent staining or flash frozen and stored in −80°C for Western blot analysis or RNA analysis. All animal studies were conducted in accordance with an institutionally approved protocol. The protocol was approved by the Institutional Animal Care and Use Committee at Rutgers, the State University of New Jersey (Protocol Number: 04-001). All surgery was performed under ketamine anesthesia, and all efforts were made to minimize suffering.

### Statistical Analysis

Statistical significance was evaluated using the Student’s *t* test.

## Results

### 1α,25(OH)_2_D_3_ and Gemini Vitamin D Analog BXL0124 Inhibit Cell Proliferation, Metabolic Activity and Invasion of MCF10DCIS Cells

**Figure 1:**
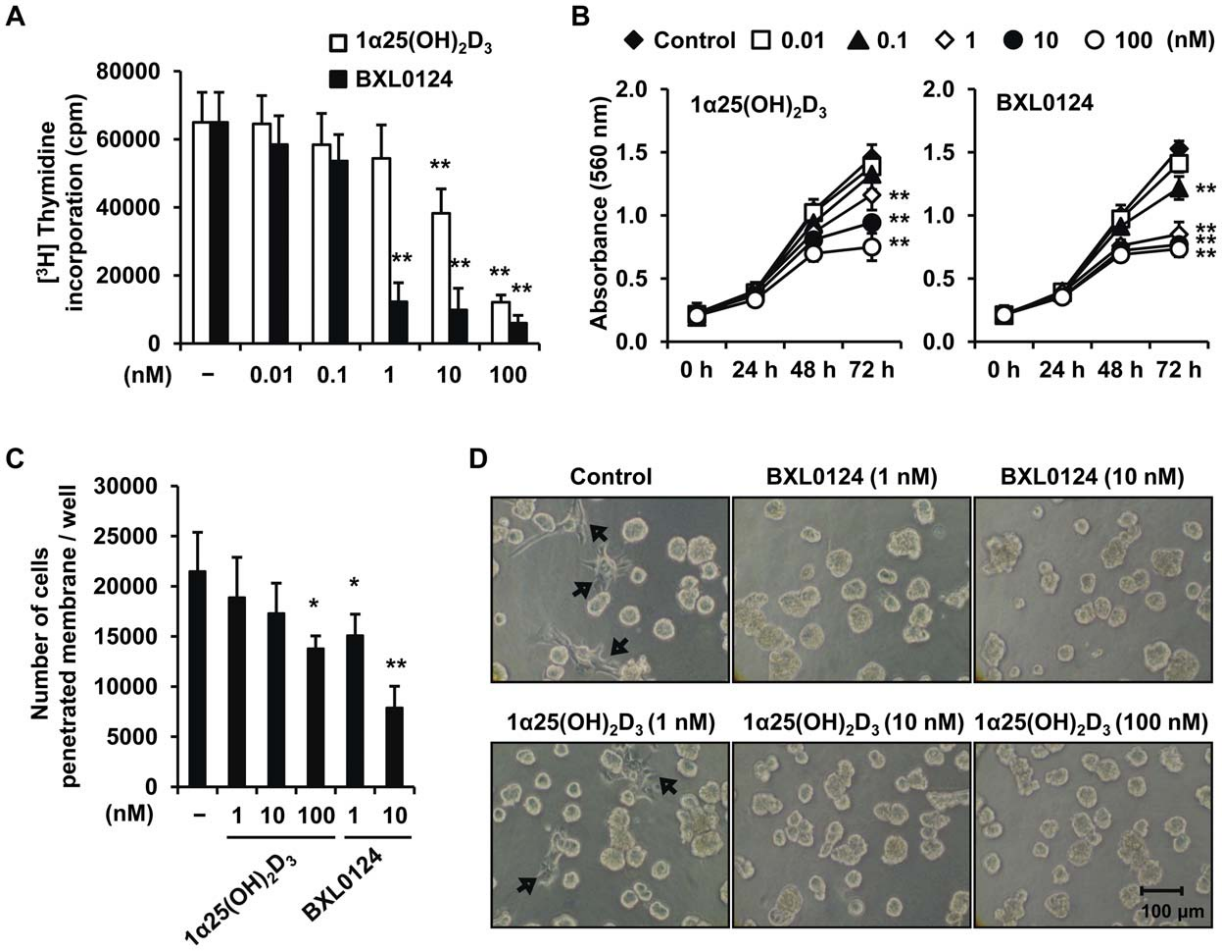
1α,25(OH)_2_D_3_ and Gemini vitamin D analog BXL0124 repress proliferation, metabolic activity and invasion of MCF10DCIS breast cancer cells. MCF10DCIS cells were incubated with 0.01, 0.1, 1, 10 or 100 nM of 1α,25(OH)_2_D_3_ or the Gemini vitamin D analog BXL0124 for 72 h. (A) The cell proliferation of MCF10DCIS cells was measured by thymidine incorporation rate. Two separate experiments with triplicates were conducted (*p<0.05, **p<0.01). (B) The metabolic activities of MCF10DCIS cells were determined by the MTT assay. Two separate experiments with quadruplicates were conducted (*p<0.05, **p<0.01). (C) MCF10DCIS cells were incubated in the basement membrane extract (BME)-coated invasion chambers in the presence or absence of 1α,25(OH)_2_D_3_ (1, 10 or 100 nM) or BXL0124 treatment (1 or 10 nM) for 48 h. The cells that penetrated through BME layer were detected from the bottom of chamber, and counted using Calcein-AM staining. Two separate experiments with triplicates were conducted (*p<0.05, **p<0.01). (D) MCF10DCIS cells were incubated in 3D culture with or without 1α,25(OH)_2_D_3_ (1, 10 or 100 nM) or BXL0124 (1 or 10 nM) for 10 days, with replenishing medium every 2 days. Representative images are shown, and the cells with invasive outgrowth are indicated with arrows.

We investigated the potential inhibitory effects of 1α,25(OH)_2_D_3_ or BXL0124 on proliferation, metabolic activity, and invasion of MCF10DCIS cells. Both 1α,25(OH)_2_D_3_ and BXL0124 significantly inhibited MCF10DCIS cell proliferation and metabolic activity (Figs. 1A and 1B, respectively); BXL0124 was more potent than 1α,25(OH)_2_D_3_. Both 1α,25(OH)_2_D_3_ and BXL0124 significantly decreased the number of MCF10DCIS cells that penetrated BME-coated layers. However, BXL0124 was more effective than 1α,25(OH)_2_D_3_ to repress MCF10DCIS cell invasion (Fig. 1C). In the 3D culture, MCF10DCIS cells showed invasive outgrowth at Day 10 (Fig. 1D, arrows), which was not detected when the cells were treated with BXL0124 (1 and 10 nM) or 1α,25(OH)_2_D_3_ (10 and 100 nM) (Fig. 1D).

### Gemini Vitamin D Analog BXL0124 Represses the Expression Levels of Invasion Markers and STAT3 Signaling of MCF10DCIS Cells

**Figure 2:**
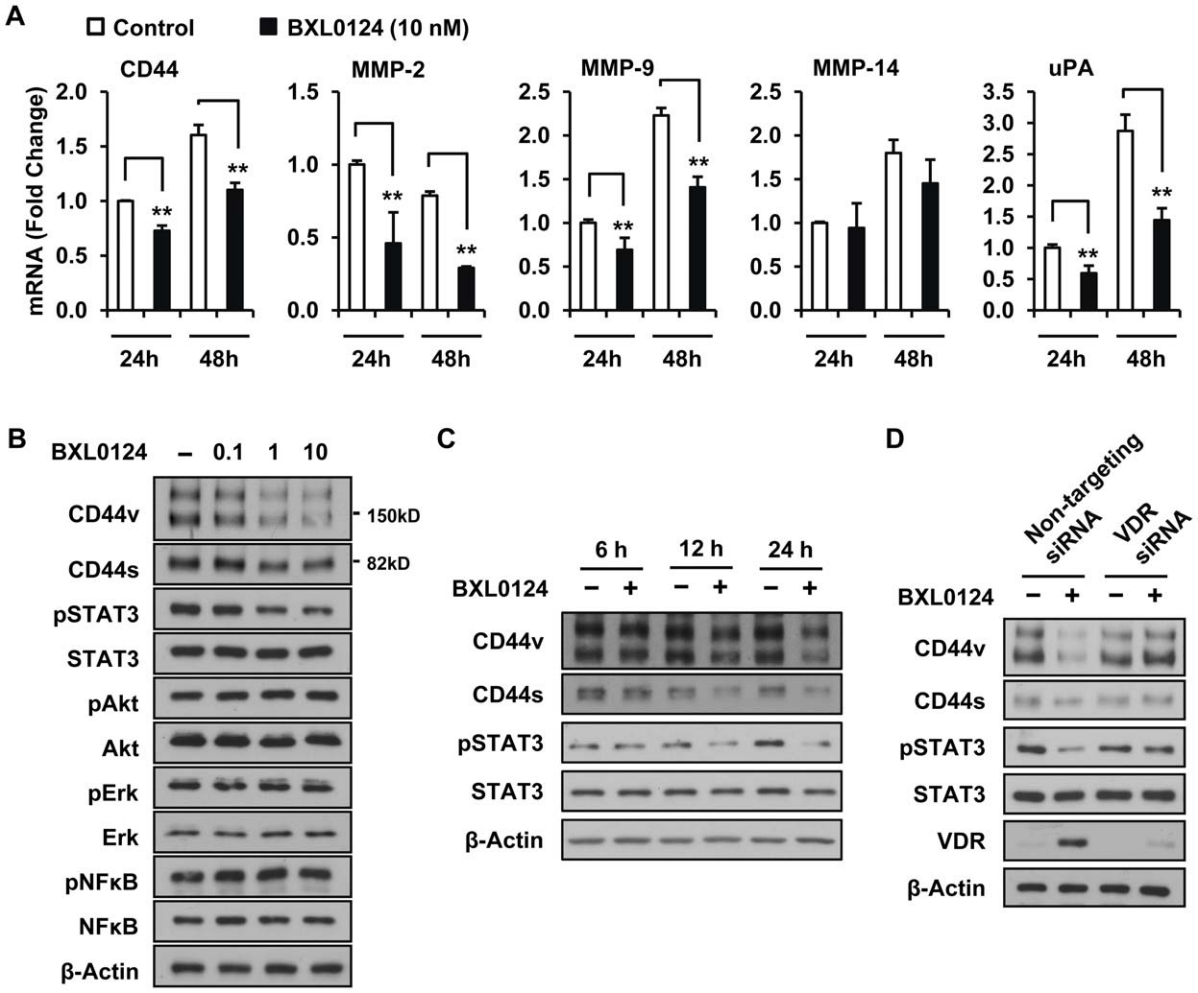
BXL0124 represses invasion markers and decreases protein levels of CD44 and pSTAT3 in MCF10DCIS cells. (A) MCF10DCIS cells were treated with BXL0124 (10 nM) for 24 h and 48 h. The mRNA expression levels of CD44 (20, the approximate qPCR cycle number of 24-h control), MMP-2 (24), MMP-9 (29), MMP-14 (23) and uPA (21) were determined. Three separate experiments with duplicates were conducted (*p<0.05, **p<0.01). (B) MCF10DCIS cells were treated with BX0124 (0.1, 1 or 10 nM) for 24 h. (C) MCF10DCIS cells were treated with BXL0124 (10 nM) for 6 h, 12 h and 24 h. (D) MCF10DCIS cells were transfected with non-targeting siRNA or VDR siRNA and treated with BXL0124 (10 nM) for 24 h. The protein levels of indicated molecules were examined by Western blot analysis, and β-actin was used as a loading control.

The mRNA expression levels of CD44, MMP-2, MMP-9, MMP-13, MMP-14, MMP-15, MMP-16, TIMP-1, TIMP-2 and uPA were investigated to identify the invasion markers regulated by BXL0124 in MCF10DCIS cells. The mRNA expression levels of CD44, MMP-2, MMP-9, and uPA were significantly decreased by BXL0124 treatment at 24 h and 48 h (Fig. 2A); MMP-14 (Fig. 2A) and other invasion markers (data not shown) did not show significant changes. To identify downstream signaling pathways that may be affected by BXL0124, the protein levels of CD44, as well as potential downstream signaling molecules (pAkt, pErk, pSTAT3 and NFκB), were measured. The BXL0124 treatment decreased the protein levels of variant isoforms of CD44 (CD44v, 100∼250 kDa), standard isoform of CD44 (CD44s, 85 kDa) and pSTAT3 in a dose-dependent manner, whereas the protein levels of pErk, pAkt and pNFκB were not changed (Fig. 2B). Total protein levels of STAT3, Akt, Erk and NFκB were not affected by the BXL0124 treatment (Fig. 2B). In a time-dependent study, the treatment with BXL0124 decreased the protein levels of CD44s and CD44v as well as pSTAT3 at 12 h and 24 h, while there was no change in the level of STAT3 (Fig. 2C). The repression of CD44 and pSTAT3 protein levels shown by the treatment with BXL0124 was abolished by knockdown of VDR using VDR siRNA, indicating that the repression of CD44-STAT3 signaling by BXL0124 is a VDR-dependent event (Fig. 2D).

### BXL0124 Inhibits Activation of STAT3 Signaling by Reducing the Complex Formation of CD44, STAT3 and JAK2

**Figure 3:**
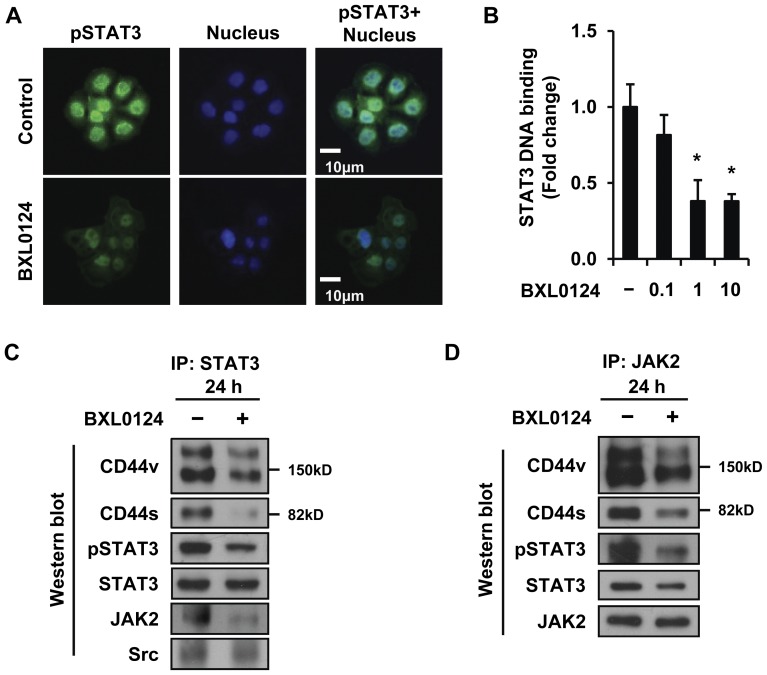
BXL0124 inhibits STAT3 activation and CD44-STAT3 interaction in MCF10DCIS cells. (A) MCF10DCIS cells were treated with BXL0124 (10 nM) for 24 h. Cells were fixed using 4% paraformaldehyde and stained with antibody against pSTAT3 (green). Nuclei were stained with To-PRO-3 (blue). (B) MCF10DCIS cells were treated with BXL0124 (0.1, 1 or 10 nM) for 24 h. Each cell lysate was incubated with oligonucleotides containing STAT3 binding sequences. The amount of STAT3 bound to the oligonucleotides was measured as chemiluminescent intensity value by luminometer. The fold change of chemiluminescent intensity value in each sample from control was determined (*p<0.05). (C) and (D) MCF10DCIS cells were treated with BXL0124 (10 nM) for 24 h, then the cell lysates were immunoprecipitated with STAT3 or JAK2 antibodies. The amounts of given proteins interacting with STAT3 or JAK2 were determined by Western blot analysis. STAT3 and JAK2 were used as loading control for each immunoprecipitation experiment, respectively.

To determine STAT3 activity affected by BXL0124, nuclear localization and DNA binding activity of STAT3 were analyzed. Strong nuclear staining of pSTAT3 was evident in the control; it was reduced by treatment with BXL0124 (Fig. 3A). DNA binding of STAT3 was also significantly decreased in a dose-dependent manner by BXL0124 treatment for 24 h (Fig. 3B). Since BXL0124 decreased the protein levels of CD44 and inhibited activation of STAT3 signaling, we investigated whether CD44 activates STAT3 signaling by direct interaction. When MCF10DCIS cell lysates were immunoprecipitated with STAT3 antibody, the immunocomplex contained CD44s, CD44v and JAK2, and BXL0124 decreased the amounts of CD44v and CD44s proteins interacting with STAT3 (Fig. 3C). In addition, the protein level of pSTAT3, but not STAT3, in the complex was decreased by the treatment with BXL0124 (Fig. 3C). Since CD44 does not have kinase activity, JAK2 and Src were examined as the possible intracellular kinases required for the phosphorylation of STAT3 in the CD44-STAT3 complex. JAK2 was recruited by STAT3, and the amount of JAK2 proteins interacting with STAT3 was decreased with the BXL0124 treatment (Fig. 3C). Src was pulled down with STAT3, but the interaction was not changed by BXL0124 (Fig. 3C). When MCF10DCIS cell lysates were immunoprecipitated with JAK2 antibody, significant amounts of CD44 and STAT3 were pulled down in the complex. This suggests that JAK2 forms a complex with CD44 and STAT3. The BXL0124 treatment decreased the amount of CD44v, CD44s, and pSTAT3 proteins interacting with JAK2 while the JAK2 level remained constant (Fig. 3D).

### CD44 Knockdown Significantly Decreases mRNA Expression Levels of Invasion Markers, MMP-9, MMP-14 and uPA, as well as MCF10DCIS Cell Invasion

**Figure 4:**
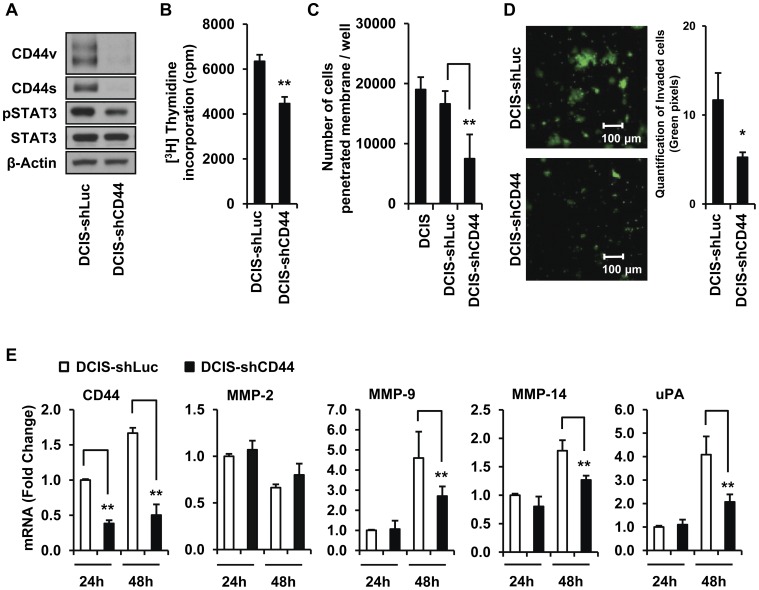
CD44 knockdown inhibits cell invasion and down-regulates MMP-9, MMP-14 and uPA. (A) The protein levels of CD44v, CD44s and pSTAT3 were markedly repressed in DCIS-shCD44 cells. (B) DCIS-shLuc or DCIS-shCD44 cells (2,000 cells/well) were incubated for 48 h and cell proliferation was determined by thymidine incorporation. Two separate experiments with triplicates were conducted (** p<0.01). (C) MCF10DCIS (DCIS), DCIS-shLuc or DCIS-shCD44 cells were incubated for 48 h in BME-coated invasion assay chambers. The number of cells that penetrated the BME layer was counted by Calcein-AM staining. Two separate experiments with triplicates were conducted (**p<0.01). (D) DCIS-shLuc or DCIS-shCD44 cells were incubated for 48 h in Fluoroblok biocoat invasion assay chambers. Since both cells were labeled with green fluorescence, the cells that penetrated matrigel layer were detected as green pixels in the image. The green pixels were counted using Image-J program for quantitative evaluation. Two separate experiments with triplicates were conducted (*p<0.05). (E) The mRNA expression levels of CD44 (20, the approximate qPCR cycle number of DCIS-shLuc cells at 24 h), MMP-2 (24), MMP-9 (29), MMP-14 (23) and uPA (21) in DCIS-shLuc and DCIS-shCD44 cells were determined after 24 h and 48 h of incubation. Three separate experiments with duplicates were conducted (**p<0.01).

To investigate the role of CD44 on DCIS invasion, we used CD44-knockdown MCF10DCIS cells transduced with shRNA for CD44 (DCIS-shCD44) or Luciferase (DCIS-shLuc) as a control. Western blot analysis showed decreased protein levels of CD44 and pSTAT3 in DCIS-shCD44 cells (Fig. 4A). Knockdown of CD44 significantly decreased the proliferation of MCF10DCIS cells (Fig. 4B). As shown in Fig. 4C, the invasive potential of MCF10DCIS cells without lentivirus infection (DCIS) or DCIS-shLuc cells, demonstrated by using the BME-coated chamber assay, was not significantly different. However, the invasive potential of DCIS-shCD44 cells was significantly decreased (Fig. 4C). To confirm the finding, we used Fluoroblok Biocoat cell invasion assay chambers with a fluorescence-blocking bottom membrane that detects only cells capable of migrating through matrigel. Because DCIS-shLuc and DCIS-shCD44 cells were transduced with shRNA constructs containing green fluorescent protein (GFP), the green fluorescent cells that penetrated through matrigel were detected at the bottom of the chamber and quantified by counting green pixels (Fig. 4D). The knockdown of CD44 significantly inhibited invasive potential of MCF10DCIS cells (Fig. 4D). CD44 mRNA expression was significantly decreased in the DCIS-shCD44 cells at both 24 h and 48 h, confirming the knockdown of CD44 by shRNA (Fig. 4E). We further determined the invasion markers that are changed by the knockdown of CD44. The mRNA expression levels of MMP-2, MMP-9 MMP-13, MMP-14, MMP-15, MMP-16, and uPA at 24 h and 48 h in DCIS-shCD44 cells were compared to those in DCIS-shLuc cells. The mRNA expression of MMP-9, MMP-14 and uPA was significantly lower in the DCIS-shCD44 cells than in the DCIS-shLuc control cells at 48 h (Fig. 4E); MMP-2 (Fig. 4E) and other invasion markers (data not shown) did not show significant changes.

### CD44 Knockdown Decreases Tumor Growth and Weight as well as Invasion Markers of MCF10DCIS Xenograft Tumors

**Figure 5:**
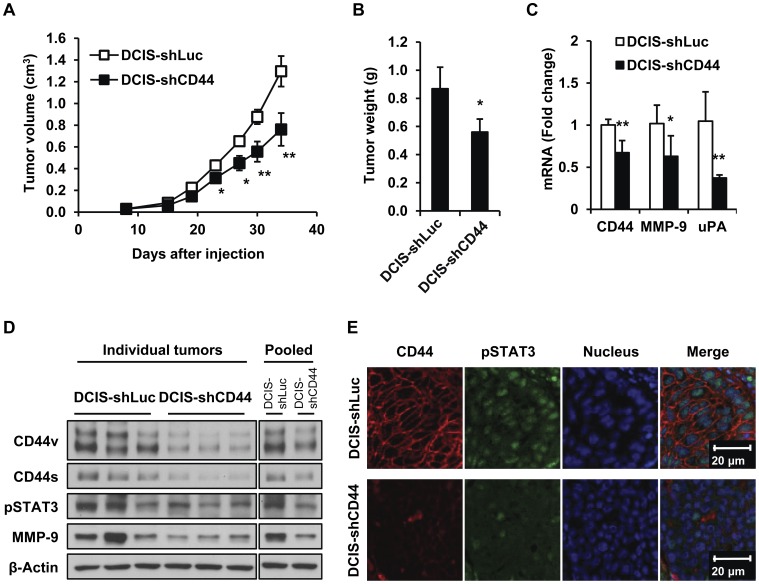
CD44 knockdown inhibits MCF10DCIS xenograft tumor growth and invasion marker expression. DCIS-shLuc or DCIS-shCD44 cells (1.0×10^6^ cells) were injected into the mammary fat pad of nu/nu mice (n = 5 per group), and mammary tumor size was measured twice a week. (A) The xenograft tumors from DCIS-shCD44 cells showed significantly slower growth rate than that of DCIS-shLuc xenograft tumors (*p<0.05, **p<0.01). (B) The average tumor weight from DCIS-shCD44 cells was significantly smaller than that from DCIS-shLuc cells (*p<0.05). (C) The mRNA expression levels of CD44 (21, the approximate qPCR cycle number of DCIS-shLuc tumors), MMP-9 (22) and uPA (22) were significantly down-regulated in DCIS-shCD44 xenograft tumors (n = 5) (*p<0.05, **p<0.01). (D) The protein levels of CD44v, CD44s, pSTAT3, and MMP-9 were markedly decreased in DCIS-shCD44 xenograft tumors. Three xenograft tumors from each group were combined for pooled samples. β-Actin was used as a loading control. (E) The protein levels of CD44 and pSTAT3 in DCIS-shLuc and DCIS-shCD44 xenograft tumors were determined by immunofluorescent staining of CD44 (red) and pSTAT3 (green). Nuclei were stained with TO-PRO-3 (blue).

To determine the role of CD44 *in vivo*, DCIS-shLuc and DCIS-shCD44 cells were injected into nu/nu mice, and tumor growth was compared. The growth rate of DCIS-shCD44 xenograft tumors was significantly slower than that of DCIS-shLuc control xenograft tumors (Fig. 5A). The average tumor weight from DCIS-shCD44 xenograft (560±93 mg) was significantly lower than that from DCIS-shLuc xenograft (870±150 mg) (p<0.05) (Fig. 5B). The levels of CD44 mRNA and protein were significantly lower in the xenograft tumors from DCIS-shCD44 cells 5 weeks after the cell injection, indicating stable knockdown of CD44 (Figs. 5C and 5D). In addition, the mRNA expression levels of MMP-9 and uPA were significantly lower in DCIS-shCD44 xenograft tumors compared to those in DCIS-shLuc xenograft tumors (Fig. 5C). The protein levels of CD44v, CD44s, pSTAT3 and MMP-9 were markedly low in the DCIS-shCD44 xenograft tumors (Fig. 5D). Immunofluorescence staining confirmed the decreased levels of CD44 and pSTAT3 in DCIS-shCD44 xenograft tumors compared to DCIS-shLuc xenograft tumors (Fig. 5E).

### BXL0124 Represses the Protein Expression Levels of CD44 and pSTAT3 in MCF10CA1a and MDA-MB-468 Basal-like Breast Cancer Cells

**Figure 6:**
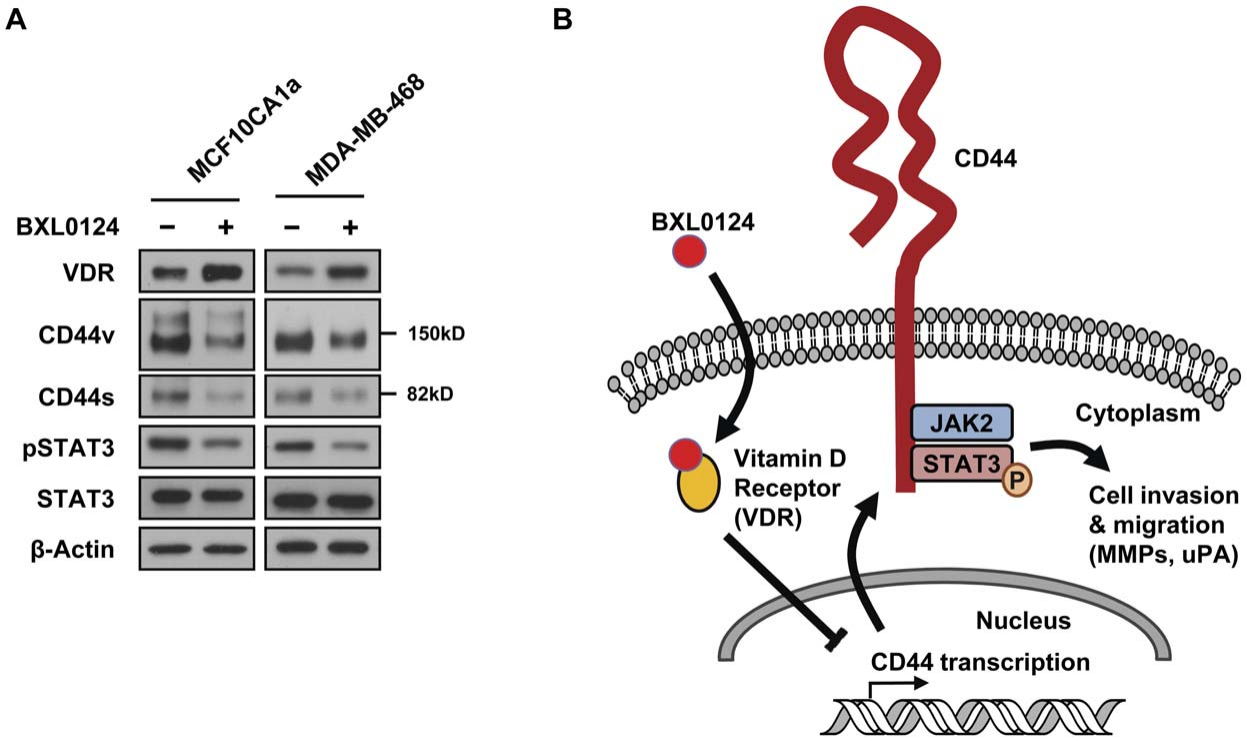
The repression of CD44-STAT3 signaling by BXL0124 in basal-like breast cancer cells. (A) MCF10CA1a and MDA-MB-468 cells were incubated with BXL0124 (10 nM) for 24 h and the protein levels of VDR, CD44v, CD44s, pSTAT3 and STAT3 were determined by Western blot analysis. β-Actin was used as a loading control. (B) A schematic diagram of proposed mechanism of action of BXL0124 on CD44-STAT3 signaling and breast cancer cell invasion in basal-like breast cancer.

To confirm the inhibitory effect of BXL0124 on CD44-STAT3 signaling in other basal-like breast cancer cells, MCF10CA1a and MDA-MB-468 cells were tested. Both MCF10CA1a and MDA-MB-468 cells showed a markedly higher expression level of CD44v than CD44s, an expression pattern similar to that for CD44 in MCF10DCIS cells (Fig. 6A). The protein level of VDR was increased by the BXL0124 treatment (Fig. 6A). BXL0124 decreased the protein levels of CD44v, CD44s and pSTAT3, whereas the protein level of total STAT3 was not affected (Fig. 6A).

## Discussion

MCF10DCIS cells form DCIS-like lesions which spontaneously progress to invasive ductal carcinoma (IDC) in immunodeficient mice [24]. The genetic alterations as well as expression patterns of molecular markers in the MCF10DCIS model greatly resemble human DCIS [6]. In addition, with the unique bipotential progenitor property, MCF10DCIS cells give rise to not only epithelial cells but also to myoepithelial cells, which represent a critical component of the DCIS to IDC transition [6], [30]. Therefore, MCF10DCIS cells can serve as a unique tool to investigate preventive therapeutics that block or delay the progression from DCIS to IDC. Recently, Jedeszko *et al.* showed that invasion of MCF10DCIS cells was significantly increased by recombinant hepatocyte growth factor (HGF). The authors also identified increased expressions of uPA and uPAR as a critical cellular response to HGF in the increased invasion [31]. Moreover, the coinjection of HGF-secreting fibroblast increased the invasiveness of MCF10DCIS xenograft tumors, promoting the transition of DCIS to IDC in immunodeficient mice [31]. In the present study, BXL0124 significantly decreased proliferation and invasion markers in MCF10DCIS cells, suggesting that BXL0124 may be an important preventive agent delaying the transition of DCIS to IDC.

CD44 is overexpressed in many cancers and is involved in malignant tumor progression as well as metastasis [8]. A recent study by Montgomery *et al*. demonstrated that knockdown of CD44 repressed both basal and hyaluronan-induced invasion of basal-like breast cancer cells [32]. In the present study, we found that repression of CD44 by BXL0124 (Figs. 1 and 2) or CD44-shRNA (Figs. 4 and 5) significantly decreased the invasive potential of MCF10DCIS cells. Furthermore, we identified STAT3 as a downstream target of CD44 in MCF10DCIS invasion (Figs. 2 and 3). In mouse mammary tumor cells, knockdown of STAT3 strongly inhibits tumor invasion without affecting cell proliferation [19], supporting the notion of a specific role of CD44-STAT3 signaling in cancer cell invasion.

Hyaluronan stimulates the interaction between CD44 and Nanog, an embryonic stem cell transcription factor, leading to activation of STAT3, and knockdown of STAT3 by siRNA blocks hyaluronan-induced breast cancer cell growth [33]. In colon cancer cells, CD44 translocates into nucleus and directly interacts with STAT3 in response to osteopontin [34]. Moreover, ectopic expression of CD44 markedly increased STAT3 activation, indicating a direct regulation of STAT3 signaling by CD44 [34]. In the present study, MCF10DCIS cells showed high CD44 protein level and constitutively activated STAT3 signal (Fig. 2B). In MCF10DCIS cells, CD44 interacts with STAT3 in the absence of exogenous ligands, suggesting that a constitutively high level of CD44 might be sufficient to activate STAT3 signaling for cell invasion. In addition, STAT3 and JAK2 interaction was decreased with BXL0124-induced repression of the CD44 (Fig. 3C and 3D), indicating that CD44 might function as a scaffold protein for the CD44-STAT3-JAK2 complex. The JAK2/STAT3 signaling pathway is preferentially activated in CD44^+^ breast cancer stem cell population over other cell populations, and hyaluronic acid synthase 1 (HAS1) is a STAT3 signaling-related molecule in basal-like breast cancer [21]. In addition, recent studies reported that STAT3 is one of the key signaling molecules that maintain breast cancer stem cell population [35], and that knockdown of STAT3 with shRNA markedly repressed mammary tumorigenesis in mice [19]. As summarized in Fig. 6B, the direct interaction between CD44, STAT3 and JAK2 may be critical for activation of STAT3 in MCF10DCIS cells, and CD44 might function as a scaffold of the STAT3-JAK2 complex.

Gene regulation by STAT3 was mediated by binding of STAT3 onto the STAT-binding element with the consensus sequences [36]. STAT3 regulates a wide range of genes associated with cancer cell invasion and metastasis, and MMPs is a family of the critical STAT3 target genes [37], [38]. MMP-2, which has active STAT-binding sites with consensus sequences at the promoter region, has been identified as one of the key STAT3-regulated genes promoting tumor invasion and metastasis [39]. Potential STAT-binding sites with consensus sequences were also found in the MMP-9 promoter; transduction of constitutively activated STAT3 significantly increased mRNA level of MMP-9 and induced transformation of human epithelial cells [40]. In the present study, we demonstrated that BXL0124 repressed mRNA expression levels of MMP-2, MMP-9 and MMP-14 as well as the binding of STAT3 onto oligonucleotide bearing the consensus sequence for STAT3 binding, suggesting a possible STAT3-dependent regulation of MMPs by BXL0124. In addition, mRNA levels of MMP-9 and MMP-14 as well as activation of STAT3 were significantly decreased by knockdown of CD44 in MCF10DCIS cells, indicating that MMPs might be downstream targets of CD44/STAT3 signaling in MCF10DCIS cells.

The expression level of MMP-9 has been correlated with the level of activated STAT3 in human breast cancer [40]. MMP-9 induces cancer cell invasion by degrading collagen type IV, the most abundant component of the basement membrane [41]. In breast cancer, high expression levels of MMP-9 have been associated with node metastasis and advanced tumor stage [42]. In addition, uPA is a critical enzyme for cancer cell invasion converting plasminogen into plasmin, which degrades extracellular matrix and activates multiple MMPs, including MMP-9 [43]. Breast cancer patients with high levels of uPA activity showed a significantly shorter disease-free period [43]. A recent study demonstrated significantly elevated expression of uPA in highly invasive basal-like breast cancer in a CD44-dependent manner [32], and a protein microarray study of primary breast cancer tissue found significant correlation between expression levels of uPA and STAT3 [44]. In addition, uPA was identified as a key molecule regulated by STAT3 in wound healing and cancer [45]. These reports support our results that MMP-9 and uPA via CD44-STAT3 signaling play a critical role in breast cancer invasion inhibited by BXL0124 in basal-like breast cancer.

### Conclusion

CD44 plays an essential role in the modulation of STAT3 signaling by forming a complex with STAT3 and JAK2. Consequently, high expression levels of CD44 may lead to a constitutive activation of STAT3 signaling in basal-type breast cancer. The novel Gemini vitamin D analog BXL0124 represses the expression of CD44, which results in a decreased amount of the CD44-STAT3-JAK2 complex. Our study suggests that repression of STAT3 signaling by targeting CD44 may be a key molecular mechanism of BXL0124-induced inhibition of breast cancer invasion, a critical step in cancer progression.
